# Polymorphism rs368234815 of interferon lambda 4 gene and spontaneous clearance of hepatitis C virus in haemodialysis patients: a case-control study

**DOI:** 10.1186/s12879-021-05777-6

**Published:** 2021-01-22

**Authors:** Alicja E. Grzegorzewska, Adrianna Mostowska, Monika K. Świderska, Wojciech Marcinkowski, Ireneusz Stolarek, Marek Figlerowicz, Paweł P. Jagodziński

**Affiliations:** 1 Department of Nephrology, Transplantology and Internal Diseases, Poznan University of Medical Sciences, Przybyszewskiego 49, 60-355 Poznań, Poland; 2 Department of Biochemistry and Molecular Biology, Poznan University of Medical Sciences, Święcickiego 6, 60-781 Poznań, Poland; 3 Fresenius Nephrocare Polska, Krzywa 13, 60-118 Poznań, Poland; 4 Institute of Bioorganic Chemistry, Polish Academy of Sciences, Poznań, Poland

**Keywords:** Haemodialysis, Hepatitis C virus, Interferon-λ4 gene, Spontaneous viral clearance, Transcription factors

## Abstract

**Background:**

In non-uremic subjects, *IFNL4* rs368234815 predicts HCV clearance. We investigated whether rs368234815 is associated with spontaneous HCV clearance in haemodialysis patients and whether it is a stronger predictor of HCV resolution than the *IFNL* polymorphisms already associated with HCV clearance in dialysis subjects. We also evaluated an association of rs368234815 with patients` survival and alterations in transcription factor binding sites (TFBS) caused by *IFNL* polymorphisms.

**Methods:**

Among 161 haemodialysis patients with positive anti-HCV antibodies, 68 (42.2%) spontaneously resolved HCV infection, whereas 93 remained HCV RNA positive. Patients were tested for near *IFNL3* rs12980275, *IFNL3* rs4803217, *IFNL4* rs12979860, *IFNL4* rs368234815, and near *IFNL4* rs8099917. *IFNL4* rs368234815 polymorphism (TT/TT, ΔG/TT, ΔG/ΔG) was genotyped by restriction fragment length polymorphism analysis; other *IFNL* polymorphisms - by high resolution melting curve analysis. We used the Kaplan-Meier method with the log-rank test for survival analysis. In silico analysis included the use of ENCODE TFBS ChIP-seq data, HOCOMOCO, JASPAR CORE, and CIS-BP databases, and FIMO software.

**Results:**

The probability (OR, 95%CI, P) of spontaneous HCV clearance for rs368234815 TT/TT patients was higher than for the ΔG allele carriers (2.63, 1.38–5.04, 0.003). This probability for other major homozygotes varied between 2.80, 1.45–5.43, 0.002 for rs12980275 and 2.44, 1.27–4.69, 0.007 for rs12979860. In the additive model, rs368234815 TT/TT was the strongest predictor of HCV clearance (6.38, 1.69–24.2, 0.003). Survival analysis suggested an association of the ΔG allele with mortality due to neoplasms (log-rank *P* = 0.005). The rs368234815 ∆G allele caused TFBS removal for PLAGL1.

**Conclusions:**

In haemodialysis patients, the association of rs368234815 with the spontaneous HCV clearance is better than that documented for other *IFNL3/IFNL4* polymorphisms only in the additive mode of inheritance. However, identifying the homozygosity in the variant ∆G allele of rs368234815 means a more potent prediction of persistent HCV infection in haemodialysis subjects that we observe in the case of the variant homozygosity of other tested *IFNL3*/*IFNL4* polymorphisms. Removal of PLAGL1 TFBS in subjects harbouring the rs368234815 ∆G allele may contribute to cancer susceptibility. The association of rs368234815 with cancer-related mortality needs further studies in HCV-exposed subjects.

**Supplementary Information:**

The online version contains supplementary material available at 10.1186/s12879-021-05777-6.

## Background

Since 2002, single nucleotide polymorphisms (SNPs) located in the interferon-λ genetic region (*IFNL*) on chromosome 19q13 are associated with antiviral protection [1, 2]. In humans, polymorphisms of the interferon-λ3 gene *(IFNL3)* or located near *IFNL3* (mainly rs12979860, rs4803217, rs8099917, rs12980275) became established genetic markers of spontaneous and PEGylated interferon-based antiviral drug-induced hepatitis C virus (HCV) clearance [3–8].

In 2013, Prokunina-Olsson et al. [9] discovered the interferon-λ4 gene (*IFNL4)* located on chromosome 19q13.2 upstream of *IFNL3* that harbours the dinucleotide frame-shift variant rs368234815 (TT/ΔG), named initially ss469415590. The polymorphism rs368234815, located within the first *IFNL4* exon, is classified as a deletion/insertion genetic variation (TT/ΔG). The *IFNL4* rs368234815 polymorphism controls a generation of the functional protein – interferon-λ4 (IFN-λ4) [9]. The ancestral *IFNL4* ΔG allele creates an open reading frame for IFN-λ4, whereas the alternative *IFNL4* TT variant eliminates IFN-λ4 [9]. *IFNL4* rs368234815 influences the signalling pathway between interferon-λ3 receptor 1 (IFN-λR1) and interferon-stimulated response element (ISRE). Overexpression of *IFNL4* suppresses *IFNL3* induction and promoter activation [10]. Transient *IFNL4* overexpression in a hepatoma cell line induced signal transducer and activator of transcription (STAT)1/STAT2 phosphorylation and expression of interferon-stimulated genes (ISG) [9]. Low intracellular expression of IFN-λ4 induces IFN-λ expression, leading to the Janus activated kinase (JAK)-STAT signalling and expression of ISG [11]. The favourable outcome of HCV infection correlates with the inability to encode IFN-λ4 [9, 10].

There is a high linkage disequilibrium (LD) between rs368234815 and rs12979860, previously designated *IFNL3* rs12979860 [12]. At present, we know that rs12979860 lies in the intron 1 of *IFNL4* and has to be related to *IFNL4.* Since 2013, we know that rs12979860 is associated with HCV spontaneous or therapeutic eradication due to its high LD with *IFNL4* rs368234815 (TT/ΔG) [9]. In Ferraris et al. [11], *IFNL4* ∆G showed a 100% correlation with the TT genotype of rs12979860. Compared to rs12979860, rs368234815 appeared similarly [9, 13] or more strongly [14–16] associated with HCV clearance. Unfavourable (variant) alleles of both rs12979860 and rs368234815 were also associated with reduced clearance of RNA viruses from the respiratory tract [17]. *IFNL3* rs4803217, altering *IFNL3* mRNA stability [3], is also in a substantial LD with *IFNL4* rs368234815. However, rs4803217 seems to be less strongly associated with HCV clearance *than IFNL4* rs368234815 [18].

An association between *IFNL* polymorphisms and HCV clearance was studied mainly in non-uremic subjects [4–10, 15, 16, 18, 19]. The meta-analysis by Xie et al. [20] revealed that the rs368234815 TT/TT genotype correlated with the sustained virologic response (SVR) to treatment with PEGylated interferon plus ribavirin in HCV-1/4-infected Caucasian patients but not in HCV-2/3-infected Caucasian patients. Conversely, the rs368234815 ΔG/ΔG genotype was linked to treatment failure in Caucasian patients, regardless of the HCV genotype. The rs368234815 ΔG allele was also a negative predictor of efficacy of direct-acting antivirals applied in HCV patients [21–23].

Reports concerning mentioned above associations in patients suffering from haemodialysis (HD)-dependent end-stage renal disease (ESRD) are scarce [24–27], and, to our knowledge, were not conducted for *IFNL4* rs368234815, currently designated as the most promising predictor of HCV resolution [18]. Uremic milieu changes the expression of genes [28]. Therefore, it is worthy of investigating whether the impact of *IFNL* polymorphic variants on HCV clearance is comparable in HD patients to that observed in non-uremic populations.

Our study’s primary purpose was to recognize the *IFNL3/IFNL4* impact on spontaneous HCV elimination in HD patients with particular attention paid to *IFNL4* rs368234815 polymorphism functionally associated with the outcome of HCV infection [9]. Firstly, we aimed to investigate whether *IFNL4* rs368234815 is associated with spontaneous HCV clearance in ESRD patients on regular HD treatment. If so, whether genotyping of rs368234815 in HD subjects can be more useful as a predictor of spontaneous HCV resolution than other tested *IFNL3/IFNL4* polymorphisms (near *IFNL3* rs12980275, *IFNL3* rs4803217, *IFNL4* rs12979860, near *IFNL4* rs8099917). We also planned to check whether an analysis of haplotypes of tested *IFNL3/IFNL4* SNPs could be useful in predicting HCV infection outcome in HD patients. Additionally, a retrospective survival analysis was performed concerning *IFNL4* rs368234815 polymorphism. For a better understanding of genetic mechanisms underlying differences in analysed phenotypes possible attributed to *IFNL* (spontaneous HCV clearance, survival), we used in silico methods for prediction of alterations in transcription factor (TF) binding sites (TFBS) caused by the tested polymorphisms.

## Patients and methods

### Patients

The study included 161 HD subjects with persistently positive anti-HCV antibodies. They were enrolled between January 2009 and November 2018 in the Wielkopolska region of Poland. The same patients were also tested in this study for near *IFNL3* rs12980275, *IFNL3* rs4803217, *IFNL4* rs12979860, and near *IFNL4* rs8099917. Some of them were included in our previous studies indicating associations of these SNPs with spontaneous HCV clearance in HD subjects [24, 26]. We used the current data of rs12980275, rs4803217, rs12979860, and rs8099917 to compare the predictability of spontaneous HCV clearance using the newly tested rs368234815 and previously established genetic markers of HCV resolution.

Enrolled patients were never treated with anti-HCV medications. Among them, 68 (42.2%) spontaneously resolved HCV infection, whereas 93 (57.8%) were persistently HCV RNA positive.

Eighty (49.7%) HD subjects showed positive antibodies against core antigen (anti-HBc) of hepatitis B virus (HBV); 10 (6.2%) HD individuals presented surface HBV antigen (HBsAg) positivity; 7 (4.3%) patients were persistently HBV DNA positive. All HD patients tested negative for antibodies to the human immunodeficiency virus (anti-HIV-1/HIV-2).

We obtained demographic, clinical, and baseline laboratory data of HD patients from physicians of HD facilities.

### Survival studies

All anti-HCV positive patients genotyped for *IFNL4* rs368234815 were included in the retrospective longitudinal survival study. In this group, we analysed survival probability from the onset of renal replacement therapy (RRT) to the last data collection (1st - 10th November 2020) concerning *IFNL4* rs368234815 genotypes.

### Genotyping

Chromosomal localization of tested *IFNL3* and *IFNL4* polymorphisms is shown in Supplementary Fig. 1A. of the Additional file 1.

*IFNL4* rs368234815 polymorphism (TT/TT, ΔG/TT, ΔG/ΔG) was genotyped by a polymerase chain reaction-restriction fragment length polymorphism (PCR-RFLP) method, as recently described by Pouryasin et al. [29]. Genotyping of rs4803217, rs12980275, rs8099917, and rs12979860 polymorphisms was carried out by a high-resolution melting curve analysis (HRM) as described in our previous studies [24, 26]. Supplementary Table 1 in the Additional file 1 shows conditions for identifying polymorphisms genotyped by PCR-RFLP or HRM.

For quality control, approximately 10% of the randomly chosen samples were re-genotyped using the same genotyping method; the concordance rate was 100%. Among 161 tested samples, two (1.2%) failed the genotyping of rs12980275 and rs4803217 each, four (2.5%) – rs12979860, and one (0.6%) - rs8099917. We excluded samples that failed the genotyping from statistical analyses.

### Prediction of transcription factor binding sites

Potential regulatory impacts of the rs4803217, rs12980275, rs8099917, rs12979860, and rs368234815 through modifications of the TFBS motifs were assessed with the experimental ENCODE TFBS ChIP-seq data [30] and in silico prediction of DNA-binding sites collected in HOCOMOCO version 9 [31], JASPAR CORE version 5.0 ALPHA 2016 [32], and CIS-BP version 1.02 [33] databases with FIMO software version 4.11.1 [34].

We performed the computational analysis on the GenBank DNA sequences (contig NT_011109.17) [35] adjacent to SNP positions. FASTA sequences, one per each SNP allele, were used as an input for the FIMO. To minimize false rates, we calculated the background file directly from input sequences with MEME-suit fasta-get-Markov script.

We analysed results via a self-developed Python script to find locations of ENCODE ChIP-seq validated TFBS and FIMO predicted motif BS overlapping SNP positions. A *p*-value < 0.0005 and q-value < 0.05 were selected as the cut-off values for reliable predictions. Motifs matched in both orientations were also analysed concerning perfect reverse complement forming, thus increasing the true positive match probability. We identified TF annotation information from the SwissProt database [36] and filtered statistically significant differentially bound hits of *Homo sapiens* motifs.

### Statistical methods

The results are presented as percentages for categorical variables or medians (range) for continuous variables because they were usually non-normally distributed as determined by the Shapiro–Wilk test.

We compared demographic, clinical, and laboratory data in anti-HCV positive HD subjects stratified by HCV RNA testing results. Continuous variables were compared using the Mann-Whitney test. The Chi-squared test or exact Fisher’s test was used for comparison of categorical variables, as appropriate.

We analysed the Hardy–Weinberg equilibrium (HWE) to compare the observed genotype frequencies to the expected ones using the Chi-square test (*P* > 0.05 with df = 1 for balance).

We tested polymorphisms for trends in association using Fisher’s test if two groups were compared or the Cochran-Armitage test if three groups were compared (P_trend_). Genotype (P_genotype_) and allelic (P_allelic_) distributions were compared between the tested groups using Pearson’s Chi-squared test or Fisher’s exact test, as appropriate.

Odds ratios (OR) and 95% confidence intervals (CIs) for OR were calculated to quantify how strongly the presence or absence of the tested allele or genotype is associated with the presence or absence of selected phenotypes of HD patients. Pearson’s Chi-squared test was used for statistical evaluation of OR. All probabilities were two-tailed. We applied logistic regression to assess the significance of the *IFNL4* genotype, among other possible determinants of HCV clearance. A ROC curve was plotted to show the AUC as a measure of the accuracy of the model.

This study’s statistical power was estimated by Quanto 1.2.4, under the “unmatched case-control” study design, with the present sample sizes and genetic effects that were observed in the study. All analyses were performed with a type I error rate of 0.05.

The survival studies were retrospectively performed from each patient’s individual RRT onset (from 15 June 1983 to 27 July 2018) to each patient’s outcome data (1st - 10th November 2020). Patients who underwent renal transplantation were analysed if they returned to haemodialysis treatment. All-cause, cardiovascular, infection-related, and neoplasm-related reasons for death were analysed. These analyses were also performed using dominant, recessive, and additive models of inheritance. Due to a relatively small number of HCV exposed patients, this analysis was planned as a preliminary one. The Kaplan-Meier method with the log-rank test was solely used to estimate differences in the cumulative proportion surviving, characterizing the genotype groups in each inheritance model.

Statistical analyses of the data mentioned above were performed using Statistica version 12 (Stat Soft, Inc., Tulsa, OK, USA). *P*-values less than 0.05 were considered significant. However, a Bonferroni correction was used for significance in detailed association analyses involving rs368234815 because such associations were not previously evaluated in HD patients.

Pair-wise LD between *IFNL3* and *IFNL4* polymorphisms was computed as both D′ and r^2^ using the genotype data from the tested sample and the Haploview 4.2 software [37] (Supplementary Fig. 1B in the Additional file 1). Haplotypes were estimated using the mentioned Haploview 4.2 software (sliding windows method) and statistically analysed if their incidence in the examined group was at least 1%. Statistical significance was assessed using the 1000-fold permutation test. Only *P*-values corrected by the test rules were considered significant.

## Results

### Patients

The characteristics of HD patients exposed to HCV are shown in Table 1. Patients showing HCV RNA positivity compared with HCV RNA negative subjects were younger at RRT onset, had longer RRT duration, and more frequent chronic glomerulonephritis as a cause of ESRD. Liver enzyme activities were higher in HCV RNA positive patients.

**Table 1:** Demographic, clinical, and laboratory data of 161 HD patients showing positive anti-HCV antibodies

Parameter	HCV RNA positive *n* = 93	HCV RNA negative *n* = 68	***P*** value^**†**^
Male gender	48 (51.6)	35 (51.5)	0.986
Age at RRT onset, years	45.8 (8.7–79.5)	53.5 (14.1–85.9)	0.004
Diabetic nephropathy	17 (18.3)	17 (25.0)	0.302
Chronic glomerulonephritis	31 (33.3)	11 (16.2)	0.014
Hypertensive nephropathy	10 (10.8)	9 (13.2)	0.630
RRT duration, years	13.4 (0–32.0)	7.0 (0.2–33.2)	0.0009
BMI, kg/m^2^	23.1 (15.2–50.4)	23.5 (16.2–37.1)	0.471
HBsAg positivity	5 (5.4)	5 (7.4)	0.744^‡^
Anti-HBc positivity	48 (51.6)	32 (47.1)	0.568
Not generating anti-HBs	16 (17.2)	17 (25.0)	0.226
Anti-HBs titre, IU/L	199.5 (0–8045)	284.0 (0–1100)	0.889
ALT, IU/L	24 (2–195)	12.4 (3–63)	0.00007
AST, IU/L	23 (8–152)	15 (8–46)	0.00001
ALP, IU/L	110 (15–647.3)	87.8 (45–803.8)	0.016
GGT, IU/L	48.5 (7–498)	27 (7–692)	0.001
C-reactive protein, mg/L	6.0 (0.2–127)	5.4 (0.3–101)	0.916
Albumin, g/dL	3.8 (1.9–4.7)	4.0 (2–4.9)	0.074

Results are presented as median and range (minimum-maximum) or the number of patients presenting the indicated parameter with the % of the total of tested patients shown in parentheses

*Abbreviations*: *ALP* alkaline phosphatase, *ALT* alanine aminotransferase, *AST* aspartate aminotransferase, *Anti-HBc* antibodies against core antigen of hepatitis B virus, *Anti-HCV* antibodies against hepatitis C virus, *BMI* body mass index, *GGT* gamma-glutamyltransferase, *HBsAg* surface antigen of hepatitis B virus, *HCV RNA* ribonucleic acid of hepatitis C virus, *HD* haemodialysis, *RRT* renal replacement therapy

Conversion factors to SI units are as follows: for alanine aminotransferase – 1 IU/L = 0.0167 μkat/L, for albumin – 1 g/dL = 10 g/L, for alkaline phosphatase – 1 IU/L = 0.0167 μkat/L, for aspartate aminotransferase – 1 IU/L = 0.0167 μkat/L, for C-reactive protein – 1 mg/L = 9.524 nmol/L, for gamma-glutamyltransferase – 1 IU/L = 0.0167 μkat/L

† Chi-squared test for qualitative variables and Mann-Whitney test for quantitative variables

^‡^Fisher’s exact test

### *IFNL4* rs368234815, other *IFNL polymorphism*s, and spontaneous HCV resolution

In all tested groups, the observed genotype frequencies were in concordance with HWE (Supplementary Table 2 in the Additional file 1).

In HCV exposed HD patients, *IFNL4* rs368234815 was significantly associated with spontaneous HCV elimination. The TT/TT genotype subjects benefited with a 2.6-fold higher frequency of spontaneous HCV clearance than patients harbouring the ∆G allele (Table 2). In the dominant model of inheritance, the association of rs368234815 with HCV infection outcome was similar to that documented for other tested *IFNL3/IFNL4* polymorphisms. In the additive model, rs368234815 TT/TT was the strongest predictor of HCV clearance. All these analyses were performed with the sample power equal to or exceeding 93% (Table 3).

**Table 2:** *IFNL4* rs368234815 polymorphic variants and HCV RNA positivity in HD patients exposed to HCV

Genotypes, MAF	HCV RNA positive patients (*n* = 93) n, % of all	HCV RNA negative patients (*n* = 68) n, % of all	Odds ratio (95% CI), *P*-value^†^
*IFNL4* rs368234815 (*n* = 161, P_*trend*_^†^ = 0.0009, P_*genotype*_^‡^ = 0.004)			
TT/TT	29 (31.2)	37 (54.4)	**Reference**
TT/∆G	49 (52.7)	28 (41.2)	2.233 (1.140–4.373), 0.02^§^
∆G/∆G	15 (16.1)	3 (4.4)	6.379 (1.685–24.16), 0.003
TT/∆G + ∆G/∆G vs TT/TT	64 (68.8)	31 (45.6)	2.634 (1.377–5.037), 0.003
∆G/∆G vs TT/TT + TT/∆G	15 (16.1)	3 (4.4)	4.167 (1.156–15.02), 0.02^§^
MAF	(0.42)	(0.25)	2.215 (1.364–3.597), 0.001

P for HWE: All: 0.527, HCV RNA positive: 0.451, HCV RNA negative: 0.419

*Abbreviations*: *HCV* hepatitis C virus, *IFNL4* – interferon-λ4 gene, *HD* haemodialysis, *MAF* minor allele frequency, *RNA* ribonucleic acid

^†^ − Cochran-Armitage trend Test; ^‡^ − Pearson’s Chi-squared test; § – not significant after the Bonferroni correction (*P* > 0.007)

**Table 3:** Associations of *IFNL3*/*IFNL4* polymorphisms with spontaneous HCV clearance in HD patients exposed to HCV

Gene^†^	rs no.	HCV RNA positive			HCV RNA negative			A difference in genotype/allele distribution between HCV RNA positive and HCV RNA negative			The probability of spontaneous HCV clearance in the dominant mode of inheritance^‡^		The probability of spontaneous HCV clearance in the additive mode of inheritance^§^	
		n	Genotype distribution, n (frequency)	MAF	n	Genotype distribution, n (frequency)	MAF	P trend^¶^	P genotype^∆^	P allelic^∆^	OR, 95% CI	*P* value^∆^, sample power	OR, 95% CI	*P* value^∆^, sample power
*IFNL3*	12980275	91	AA 25 (27.5) AG 48 (52.7) GG 18 (19.8)	0.46	68	AA 35 (51.5) AG 26 (38.2) GG 7 (10.3)	0.29	0.003	0.007	0.002	2.8, 1.45–5.43	0.002 97%	3.60, 1.31–9.91	0.011 > 99.9%
*IFNL3*	4803217	92	GG 29 (31.5) GT 48 (52.2) TT 15 (16.3)	0.42	67	GG 36 (53.7) GT 27 (40.3) TT 4 (6.0)	0.26	0.002	0.01	0.003	2.52, 1.32–4.84	0.005 95%	4.66, 1.39–15.56	0.008 > 99.9%
*IFNL4*	12979860	90	CC 29 (32.2) CT 45 (50.0) TT 16 (17.8)	0.43	67	CC 36 (53.7) CT 26 (38.8) TT 5 (7.5)	0.27	0.004	0.015	0.004	2.44, 1.27–4.69	0.007 93%	3.97, 1.30–12.14	0.012 > 99.9%
*IFNL4*	368234815	93	TT/TT 29 (31.2) TT/∆G 49 (52.7) ∆G/∆G 15 (16.1)	0.42	68	TT/TT 37 (54.4) TT/∆G 28 (41.2) ∆G/∆G 3 (4.4)	0.25	0.0009	0.004	0.001	2.63, 1.38–5.04	0.003 97%	6.38, 1.69–24.2	0.003 > 99.9%
*IFNL4*	8099917	92	TT 48 (52.2) GT 38 (41.3) GG 6 (6.5)	0.27	68	TT 51 (75) GT 17 (25) GG 0 (0)	0.13	0.001	0.003^#^	0.001	2.75, 1.39–5.45	0.003 98%	13.8, 0.76–251.6	0.027^#^ > 99.9%

† − chromosomal localization of *IFNL3* and *IFNL4* genes towards the forward strand is shown

‡ − genotypes with the variant alleles as reference

§ – the variant homozygosity as reference

¶ - Cochran-Armitage Trend Test

∆ - Pearson’s Chi-squared test

# - Fisher’s exact test

###  *IFNL4* rs368234815 as a predictor of spontaneous HCV clearance among other clinical data

**Fig. 1:**
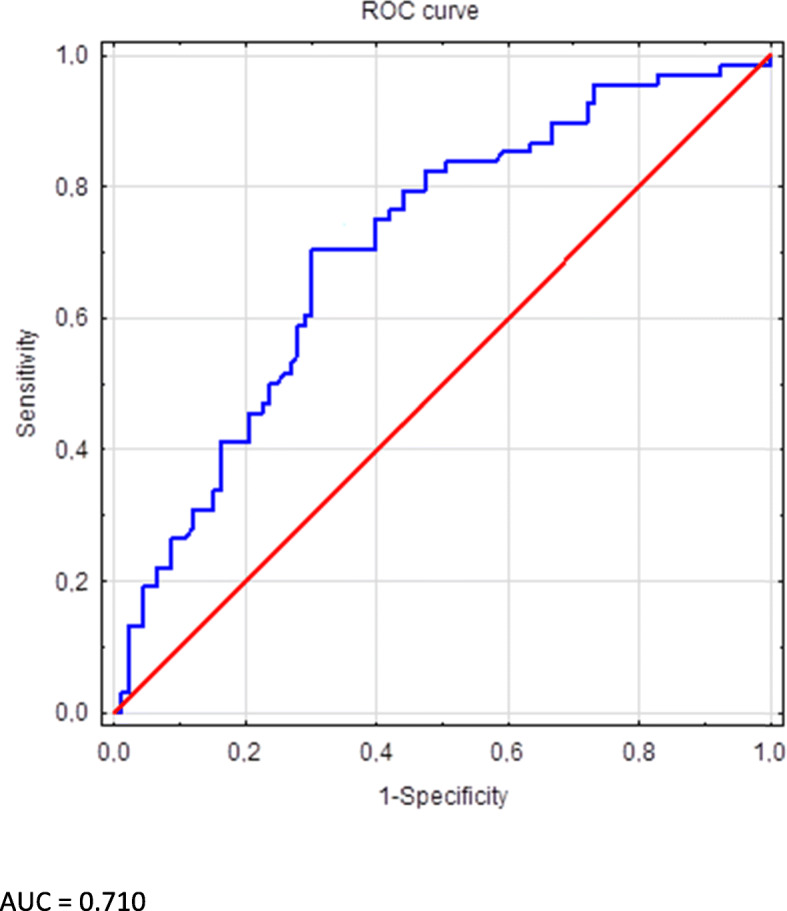
The area under the curve as a measure of the accuracy of the model created to predict spontaneous HCV clearance. A receiver operating characteristic (ROC) curve was plotted to show the area under the curve (AUC) as a measure of the accuracy of the model created for evaluation of selected explanatory variables of spontaneous HCV clearance. The model included the TT/TT genotype of rs368234815, age at RRT onset, RRT duration, and chronic glomerulonephritis. All these variables significantly differed HCV RNA positive and HCV RNA negative HD individuals (Table 1)

With the TT/TT genotype of rs368234815, age at RRT onset, RRT duration, and chronic glomerulonephritis were chosen as possible explanatory variables for HCV spontaneously clearance. Among tested variables, only the TT/TT genotype remained a significant predictor of HCV resolution (OR 2.526, 95% CI 1.274–5.009, *P* = 0.008). The accuracy of the model in the receiver operating characteristic (ROC) curve analysis indicated the area under the curve (AUC) of 0.710 (Fig. 1).

### Linkage disequilibrium

We examined LD between the rs12980275, rs4803217, rs12979860, rs368234815, and rs8099917 SNPs (Supplementary Fig. 1B in the Additional file 1). *IFNL4* rs368234815 was the most strongly associated with rs12979860 (*r*^*2*^ = 0.81); the weakest association was shown between this SNP and rs8099917 (*r*^*2*^ = 0.31).

###  *IFNL3* and *IFNL4* haplotype analysis

We analysed the haplotypes of *IFNL3* and *IFNL4* SNPs concerning the outcome of HCV infection.

Results including haplotypes of 2–4 SNPs are shown in Supplementary Table 3 in the Additional file 1. All haplotypes composed exclusively of major alleles were significantly associated with spontaneous HCV clearance.

Data concerning the haplotypes of all five tested SNPs are presented in Table 4. If all haplotypes other than the tested one were pooled together and used as the reference, spontaneous HCV clearance was positively associated with the haplotype composed of major alleles of all five tested *IFNL3*/*IFNL4* SNPs (OR 2.294, 95% CI 1.412–3.727, *P* = 0.0007). The haplotype, associated with a lower probability of spontaneous HCV resolution, was composed of variant alleles of all five tested polymorphisms (OR 0.380, 95% CI 0.197–0.732, *P* = 0.003). When the tested haplotype included both variant and major alleles of *IFNL3* and *IFNL4* SNPs, there was no significance in the outcome of HCV infection (Table 4 and Supplementary Table 3 in the Additional file 1).

**Table 4:** Frequencies of *IFNL3* and *IFNL4* haplotypes concerning spontaneous HCV elimination (cases) and persistent HCV infection (controls)

SNPs	Haplotype	Haplotype Frequencies	Case, Control Frequencies	Chi Square	P Value	P_corr_ Value^†^	OR (95%CI)^‡^, *P* Value	OR (95%CI)^§^, P Value
rs12980275_rs4803217_rs12979860_rs368234815_rs8099917	A_C_C_TT_T	0.562	0.667, 0.485	10.590	0.0011	**0.0060**	**2.294 (1.412–3.727), 0.0007**	**Reference**
	G_A_T_∆G_G	0.174	0.102, 0.227	8.543	0.0035	**0.0190**	**0.380 (0.197–0.732), 0.0030**	**0.326 (0.167–0.638), 0.0008**
	G_A_T_∆G_T	0.121	0.110, 0.130	0.289	0.5909	1.0000	0.819 (0.411–1.632), 0.5692	0.611 (0.301–1.241), 0.1706
	G_C_C_TT_T	0.043	0.037, 0.046	0.164	0.6855	1.0000	0.736 (0.241–2.253), 0.5901	0.543 (0.175–1.685), 0.2847
	A_A_T_∆G_T	0.020	0.008, 0.028	1.583	0.2083	0.7840	0.263 (0.030–2.280), 0.1934	0.196 (0.022–1.709), 0.1024
	G_A_T_TT_G	0.016	0.015, 0.016	0.013	0.9081	1.0000	0.894 (0.147–5.434), 0.9032	0.652 (0.106–3.997), 0.6415

^†^*P*-value calculated using the permutation test and a total of 1000 permutations

^‡^All other haplotypes pooled together were used as the reference

^§^The most common haplotype was used as the reference

### In silico analysis

To study the potential regulatory impacts of the rs4803217, rs12980275, rs8099917, rs12979860, and rs368234815, we analysed the modifications of the TFBS motifs caused by these SNPs. Data of the in silico analysis are summarized in Table 5. Firstly, we used the public ENCODE TFBS ChIP-seq dataset from 187 TFs tested in 125 cell lines to find peaks overlapping SNP positions. We found no peaks overlapping positions of rs4803217 and rs12980275. ENCODE ChIP-seq peaks for the ZNF263, GATA1, POLR2A, and ARID3A TFs overlapped the position of the rs8099917. Positions of the rs12979860 and rs368234815 overlapped with the EZH2 ENCODE ChIP-seq peaks. In our in silico analysis, none of the predicted ENCODE ChIP-seq peaks for the TFBS overlapped positions of these SNPs directly. Therefore, they are not shown in Table 5.

**Table 5:** Prediction of TFBS by the software FIMO for rs368234815, rs12980275, rs12979860, rs8099917, and rs4803217

SNP/allele	Transcription factor	Modification (in the presence of the minor allele)	Strand	*p*-value/q-value†	Matched sequence
rs368234815/∆G	PLAG1 like zinc finger 1	Removed	“+”	7.49e-05/0.0636	CGGGGGGCCT
rs12979860/ T	Transcription factor E2F7	Removed	“+”	2.49e-05/0.0288	GAAGGCGCGAACC
rs12979860/ T	Oxysterols receptor LXR-alpha: Retinoid X receptor alpha (Nr1h3::Rxra)	Added	“+”	6.03e-05/0.0423	TGAACCAGGGTTGAATTGC
rs12979860/ T	Oestrogen Related Receptor Alpha (ESRRA)	Removed	–	4.71e-05/0.0498	TCAGGGTCAAT
rs12979860/ T	MAX Dimerization Protein (MGA)	Added	“+”	9.32e-05/0.00347	AGGCGTGA
rs12979860/ T	T-box brain protein 1 (TBR1)	Added	“+”	5.29e-05/0.0495	AGGCGTGAAC
rs4803217/ A	Transcription factor HES-2	Added	“+”	7.76e-05/0.00974	TTAAGACAAGTGG
rs4803217/ A	DNA (cytosine-5)-methyltransferase 1 (DNMT1)	Removed	“+”	9.7e-06/0.0499	CCCCGCTGGC
rs4803217/ A	Homeobox protein NKX3–2	Removed	“+”	7.51e-05/0.0107	AGCCAAGTGGC
rs4803217/ A	POU domain, class 6, transcription factor 1 (POU6F1)	Removed	“+”	4.41e-05/0.0279	AATAAATTAAGCC

*Abbreviation*: *TFBS* transcription factor binding sites

The table contains only statistically significant in silico predicted differentially bound transcription factors

† − The calculated log-odds scores were converted with a dynamic programming algorithm into *p*-values by FIMO software. The *p*-values for each motif occurrence were converted to *q*-values following Benjamini and Hochberg [38]

The computational analysis revealed no direct differential motif binding for rs12980275 and rs8099917. Differentially bound transcription factors (TFs) were revealed for rs4803217 [HES2, DNA (cytosine-5)-methyltransferase 1 (DNMT1), Homeobox protein NKX3–2, POU domain, class 6, transcription factor 1 (POU6F1)], rs12979860 [E2F7, Oxysterols receptor LXR-alpha: Retinoid X receptor alpha (Nr1h3::Rxra), MAX Dimerization Protein (MGA), Oestrogen Related Receptor Alpha (ESRRA), T-box brain protein 1 (TBR1)], and rs368234815 (PLAG1 like zinc finger 1 identified also as M6422_1.02) (Table 5).

### Survival and  *IFNL4* rs368234815

During the analysed period (an RRT duration), lasting in individual cases from 0.19 years to 34.0 years (median 11.2 years), we recorded 96 all-cause deaths of the examined HD patients. Cardiovascular, infection-related, neoplasm-related, and other reasons of deaths comprised 55.2% (*n* = 53), 15.6% (*n* = 15), 9.4% (*n* = 9), and 19.8% (*n* = 19) of all deceased patients, respectively. There were no significant differences in overall survival probability and specific reasons for death, but the neoplasm-related cause (log-rank *P* = 0.005 in the recessive inheritance mode for rs368234815, Supplementary Fig. 2 in the Additional file 1).

To further explore a possible association between the *IFNL4* rs368234815 ∆G/∆G genotype and mortality due to neoplasm, we genotyped not HCV-exposed HD patients who died from cancer in the analysed period, and their DNA samples were stored. We gathered 82 samples (*P*-value for HWE concerning rs368234815 SNP = 0.803). In this group, the ∆G/∆G genotype was not associated with mortality due to cancer disease (log-rank *P* > 0.05 for all three inheritance models).

## Discussion

*IFNL4* rs368234815 polymorphism is recognized as the most potent predictor of HCV infection outcome in the general population [18]. To our knowledge, this paper, for the first time, describes associations between *IFNL4* rs368234815 polymorphism and spontaneous HCV clearance in HD patients who substantially differ from the general population due to persistent uremic status and altered immune competence [39]. In this study, we applied the PCR_RFLP method for genotyping of rs368234815 polymorphism, which was recently described by Pouryasin et al. [29]. Among Iranian non-uremic patients with chronic HCV infection tested with this method, the rs368234815 ΔG/ΔG frequency was 17.3% [29]. Caucasian HD patients of Polish origin with persistently positive testing for HCV RNA revealed a similar frequency of the ΔG/ΔG genotype (16.1%).

Similarly, as in non-uremic subjects [14, 15], rs368234815 was associated with spontaneous HCV clearance also in HD patients. On the same group of anti-HCV positive HD patients, we were able to show that major homozygosity in rs368234815 (TT/TT) similarly predicts spontaneous HCV clearance in the dominant model of inheritance as other tested *IFNL3*/*IFNL4* polymorphisms already associated with HCV clearance in HD subjects. All tested *IFNL* polymorphisms (including rs368234815) indicated a 2.4–2.8-fold higher frequency of spontaneous HCV clearance in HD patients characterized by the major homozygosity in analysed polymorphisms.

The usefulness of the *IFNL4* rs368234815 TT/TT genotype in the predictability of spontaneous HCV clearance was superior to other tested *IFNL* polymorphisms only in the additive inheritance model. In non-uremic subjects, the rs368234815 TT/TT genotype indicated 3.6-fold (Swiss Caucasians [15]) – 12-fold (American white women [14]) higher frequency of spontaneous HCV clearance compared with the ∆G/∆G genotype. In HD patients, the TT/TT genotype predicted a 6.4-fold higher probability of spontaneous HCV clearance. Therefore, uremic conditions do not seem to influence substantially *IFNL4* rs368234815 expression concerning spontaneous HCV resolution. Additionally, an identification of the homozygosity in the variant ∆G allele of rs368234815 in HD subjects means a more potent prediction of persistent HCV infection that it is observed in the case of the variant homozygosity of other tested *IFNL3*/*IFNL4* polymorphisms.

A question was raised whether the haplotype of unfavourable rs368234815 ∆G allele and favourable major allele of any other *IFNL3*/*IFNL4* SNPs could be associated with more frequent spontaneous HCV clearance. Data of O’Brien et al. [18] excluded such a possibility for the haplotype of a favourable major allele of *IFNL3* rs4803217 [40, 41] and the unfavourable *IFNL4* rs368234815 ∆G allele concerning a day 28 anti-HCV response in African American individuals. A recent study by Vergara et al. [42] demonstrated in the European ancestry group that subjects with the haplotype rs368234815∆G_rs4803221C were 1.7 times more likely to clear HCV than individuals showing rs368234815∆G_rs4803221G haplotype. Also, the haplotype rs368234815∆G_rs8099917T was associated with a 1.6 times higher frequency of HCV resolution compared to rs368234815∆G_rs8099917G haplotype. There were no differences in association analysis of these two haplotypes concerning spontaneous HCV clearance in African ancestry [42]. In the examined European HD patients, only haplotypes containing exclusively major alleles of tested *IFNL3*/*IFNL4* SNPs were associated with spontaneous HCV resolution. In HD patients, similarly to in African Americans [42], there were no significant differences in spontaneous HCV clearance frequency if haplotype was composed of rs368234815∆G and major versus variant allele of tested *IFNL3*/*IFNL4* SNPs (Supplementary Table 4 in the Additional file 1). However, a relatively small group of the examined patients could participate in a lack of significance. It has to be noted that novel SNPs were recently identified in the *IFNL* region of which rs4803221 (and already known rs8099917) appear contributors in a primary signal of association represented by *IFNL4* rs368234815 SNP [42].

Spontaneous HCV elimination is associated with the induction of virus-specific CD4^+^ and CD8^+^ T cell responses and, to a lesser extent, with the generation of neutralising antibodies targeting the HVR1 region of HCV envelope glycoprotein 2 [43]. The rs368234815 impact on HCV infection outcome is still investigated. The *IFNL4* rs368234815 TT allele, which promotes spontaneous HCV clearance, is associated with the expression of the transcript JN806227. The latter generates a prematurely terminated IFN-λ4 protein with a lack of activity [9, 10]. The *IFNL4* ∆G allele transcript produces full-length IFN-λ4, of which IFN-λ4 -70Pro is more active, while IFN-λ4-70Ser shows diminished activity. This difference depends on nonsynonymous variant *IFNL4* rs117648444 A/G (Pro70Ser) in exon 2. *IFNL4* haplotype rs368234815∆G_rs117648444G produces a more active IFN-λ4 -70Pro and is captured by rs8099917 variant (G) allele, haplotype rs368234815TT_rs117648444G corresponds to lack of active IFN-λ4, and rs368234815∆G_rs117648444A generates a less active IFN-λ4-70Ser. Both latter haplotypes are tagged by the rs8099917 T allele [9, 44]. It is suggested that one of the possible mechanisms, by which primarily intracellular IFN-λ4 deteriorates HCV elimination, is up-regulation of IFN-λR1 what impedes receptor binding of the other members of the IFN-λ family [45]. Recent analyses [46] have shown that *IFNL4* rs12979860, being a marker for rs368234815, is strongly associated with the HCV proteome’s amino acid variants. Like that concerning leucine at position 2224 of NS5A in HCV genotype 1b in patients with the rs12979860 CC genotype, some of these associations correspond to the pre-treatment viral load. The rs12979860 variant (T) allele correlated with lower pre-treatment viral load for all HCV genotypes, while the rs12979860 CC genotype was associated with higher mean viral load. Therefore, the difference in viral load could be partially related to changes in viral amino acids associating with the presence or absence of IFN-λ4 [46]. It has to be noted that the rs12979860 CC genotype was simultaneously associated with spontaneous HCV clearance and higher viral load. The latter was inversely [47] or positively [48] correlated with spontaneous HCV elimination in clinical studies. The coincidence of rs12979860, viral load, and HCV eradication needs exploration in further investigations.

We identified that minor alleles of the rs368234815, rs12979860, and rs4803217 could cause statistically significant changes in TFBS. Therefore, the individual or combined effects of those changes may contribute to the differential *IFNL3* and *IFNL4* expression. As *IFNL3*/*IFNL4* polymorphisms are well-established predictors of HCV clearance, removal or addition of the host TFBS in the presence of the minor alleles of tested polymorphisms might be at least a partial explanation of HCV persistence in the *IFNL3*/*IFNL4* minor allele bearers. Our computational analysis provides a list of candidate differentially bound TFBS in tested variants. The experimental ChIP-seq study could be applied to verify their impact exerted by the minor alleles. The following example shows how critical is the need for such verification. In silico analysis revealed that the host TFBS for DNA DNMT1 is removed in the *IFNL3* rs4803217 minor allele bearers who are predisposed to persistent HCV infection ([18, 40, 41], this study). However, CpG islands for DNMT1-induced DNA methylation were not shown in the interferon-λ genetic region [49], so removing TFBS for DNMT1 may have no clinical relevance concerning HCV clearance. Nonetheless, the proposed putative mechanistic hypothesis describing the effects of the studied SNPs on TFBS serves as an exploratory analysis based on computational studies. Moreover, the public reference dataset is an approximation of the cellular and tissue states in studied samples.

In our study, the *IFNL4* rs368234815 ∆G/∆G genotype appeared to be associated with cancer mortality in HCV-exposed HD patients. We consider this finding as a preliminary one due to a small number of HD patients who died from cancers in this group. Additionally, such a survival analysis in the examined not HCV-exposed subjects did not reveal any relationship between neoplasm-related mortality and *IFNL4* rs368234815 SNP. On the other hand, our data are following the evidence demonstrating reduced survival in rs368234815 ∆G/∆G genotype or rs12980275 GG genotype (corresponds to rs368234815 ∆G/∆G genotype) subjects with cancers [50, 51].

IFN-λ has a dual role in cancer. Direct antitumor effects of IFN-λ include inhibition of cell proliferation, promotion of cell apoptosis, and cell cycle arrest. Indirect antitumor effects of IFN-λ include immune cell activation and angiogenesis inhibition. However, new evidence indicates that IFN-λ can also promote oncogenesis [52]. *IFNL* polymorphisms may differentiate their role in cancer antitumor effects or oncogenesis. Our in silico analyses demonstrated that neoplasm-related mortality might be due to the removal of TFBS for PLAGL1 zinc finger 1 protein, which is associated with anti-proliferative activities and tumour suppression. PLAGL1 gene is often deleted or methylated and silenced in cancer cells [53]. This exciting aspect needs further elaboration.

## Conclusions

1. In HD patients, the association of *IFNL4* rs368234815 with the spontaneous HCV clearance is more potent than that documented for other *IFNL3/IFNL4* polymorphisms (*IFNL3* rs12980275, *IFNL3* rs4803217, *IFNL4* rs12979860, and near *IFNL4* rs8099917) only in the additive mode of inheritance.
2. An identification of the rs368234815 ∆G allele homozygosity means a more potent prediction of persistent HCV infection in HD subjects than observed in the case of the variant homozygosity of other tested *IFNL3*/*IFNL4* polymorphisms.
3. Removal of PLAGL1 TFBS in subjects harbouring the rs368234815 ∆G allele may contribute to cancer susceptibility.
4. The association of rs368234815 with cancer-related mortality needs further studies in HCV-exposed HD subjects.

## Supplementary Information

**Additional file 1: **Supplementary data for polymorphism rs368234815 of interferon lambda 4 gene and spontaneous clearance of hepatitis C virus in haemodialysis patients. Supplementary material contains genotyping conditions for identifying tested polymorphisms, results of Hardy–Weinberg equilibrium, and haplotype analysis. Figures show chromosomal localization of tested *IFNL3*/*IFNL4* polymorphisms and the Kaplan-Meier cumulative proportion surviving for haemodialysis patients with neoplasms.

## Data Availability

The datasets used and analysed during the current study are available from the corresponding author on reasonable request.

## Abbreviations

AUC: Area under the curve
CI: Confidence interval
DNA: Deoxyribonucleic acid
DNMT1: DNA (cytosine-5)-methyltransferase 1
ESRD: End-stage renal disease
ESRRA: Estrogen Related Receptor Alpha
HCV: Hepatitis C virus
HD: Hemodialysis
HRM: High-resolution melting curve analysis
HWE: Hardy-Weinberg equilibrium
*IFNL*: Interferon-λ genetic region
*IFNL3*: Interferon-λ3 gene
*IFNL4*: Interferon-λ4 gene
IFN-λ: Interferon-λ
INF-λR1: Interferon-λ receptor 1
ISG: Interferon-stimulated genes
ISRE: Interferon-stimulated response element
JAK: Janus activated kinase
LD: Linkage disequilibrium
MGA: MAX Dimerization Protein
mRNA: messenger ribonucleic acid
Nr1h3::Rxra: Oxysterols receptor LXR-alpha: Retinoid X receptor alpha
OR: odds ratio
PCR-RFLP: Polymerase chain reaction-restriction fragment length polymorphism
POU6F1: POU domain, class 6, transcription factor 1
ROC: Receiver operating characteristic
RNA: Ribonucleic acid
RRT: Renal replacement therapy
SNP: Single nucleotide polymorphism
STAT: Signal transducer and activator of transcription
TBR1: T-box brain protein 1
TF: Transcription factor
TFBS: Transcription factor binding sites
