# Supplementary material for: Polymorphism rs368234815 of interferon lambda 4 gene and spontaneous clearance of hepatitis C virus in haemodialysis patients: a case-control study
Source: BMC Infect Dis. 2021 Jan 22;21:102. doi: 10.1186/s12879-021-05777-6 (PMC7821534; doi:10.1186/s12879-021-05777-6)

Supplementary data for polymorphism rs368234815 of interferon lambda 4 gene and spontaneous clearance of hepatitis C virus in haemodialysis patients

Alicja E. Grzegorzewska ^Department of Nephrology, Transplantology and Internal Diseases, Poznan University of Medical Sciences, 60-355 Poznań, Przybyszewskiego 49, Poland; e-mail: alicja_grzegorzewska@yahoo.com^

Adrianna Mostowska ^Department of Biochemistry and Molecular Biology, Poznan University of Medical Sciences, 60-781 Poznań, Święcickiego 6, Poland; e-mail:^ [^amostowska@wp.pl^](mailto:amostowska@wp.pl)

Monika K. Świderska ^Department of Nephrology, Transplantology and Internal Diseases, Poznan University of Medical Sciences, 60-355 Poznań, Przybyszewskiego 49, Poland; e-mail: monika.swi@gmail.com^

Wojciech Marcinkowski ^Fresenius Nephrocare Polska, 60-118 Poznań, Krzywa 13, Poland; e-mail: Wojciech.Marcinkowski@fmc-ag.com^

Ireneusz Stolarek ^Institute of Bioorganic Chemistry, Polish Academy of Sciences, Poznań, Poland, e-mail:^ [^stolarek.ir@gmail.com^](mailto:stolarek.ir@gmail.com)

Marek Figlerowicz ^Institute of Bioorganic Chemistry, Polish Academy of Sciences, Poznań, Poland, e-mail:^ **^ibch@ibch.poznan.pl^**

Paweł P. Jagodziński ^Department of Biochemistry and Molecular Biology, Poznan University of Medical Sciences, 60-781 Poznań, Święcickiego 6, Poland; e-mail: pjagodzi@ump.edu.pl^

Supplementary Material

**Supplementary Table 1. Genotyping conditions for the identification of tested polymorphisms**

| Gene symbol | rs no. | Alleles^†^ | Primers for PCR amplification  (5’ – 3’) | Annealing temp. (°C) | PCR product length (bp) | HRM | RFLP | |
| --- | --- | --- | --- | --- | --- | --- | --- | --- |
|  |  |  |  |  |  | Melting temp. range (°C) | Restriction enzyme | Restriction fragment length (bp) |
| *IFNL4* | rs368234815^‡^ | TT/∆G | F: GACGCAGGACCCCTTGGGACAGGA  R: TCTGGGCCGCAGTGGCCGCGAGG^§^ | 67  with 5% DMSO | 226 / 227 |  | MspA1I | TT = 227  G = 175 + 51 |
| *IFNL4* | rs8099917 | G/T | F: TTTGTCACTGTTCCTCCTTTTG | 61.0 | 98 | 76 - 86 |  |  |
|  |  |  | R: AAGACATAAAAAGCCAGCTACCA |  |  |  |  |  |
| *IFNL4* | rs12979860 | C/T | F: CGTGCCTGTCGTGTACTGAA | 61.0 | 148 | 86 - 96 |  |  |
|  |  |  | R: AGGCTCAGGGTCAATCACAG |  |  |  |  |  |
| *IFNL3* | rs4803217 | A/C | F: CATAAATAGCGACTGGGTGACA  R: GACCTGTGTGTCTGACCCTTC | 65.0 | 99 | 75 – 90 |  |  |
| *IFNL3* | rs12980275 | A/G | F: CATGAGGTGCTGAGAGAAGT  R: ATTGTTCGGCAAGCAATCTC | 53 | 155 | 78 – 93 |  |  |

Abbreviations: DMSO - Dimethyl sulfoxide, HRM - High-Resolution Melt analysis, *IFNL* - interferon lambda gene, RFLP - Restriction Fragment Length Polymorphism.

^†^Underline denotes the minor allele.

^‡^Primer sequences and genotyping conditions were taken from Pouryasin et al.^29^

^§^The underlined nucleotide represents a mismatch to abolish the non-specific restriction site for MspA1I.

Supplementary Table 2. The Hardy–Weinberg equilibrium (HWE) for tested groups of anti-HCV positive patients

| Gene^†^ | rs no. | HWE P-value for all patients | HWE P-value for HCV RNA positive patients | HWE P-value for HCV RNA negative patients |
| --- | --- | --- | --- | --- |
| *IFNL3* | 12980275 | 0.784 | 0.559 | 0.514 |
| *IFNL3* | 4803217 | 0.709 | 0.513 | 0.718 |
| *IFNL4* | 12979860 | 0.817 | 0.840 | 0.919 |
| *IFNL4* | 368234815 | 0.527 | 0.451 | 0.419 |
| *IFNL4* | 8099917 | 0.628 | 0.686 | 0.239 |

^†^ The Hardy–Weinberg equilibrium was analysed using the Chi-square test (P > 0.05 with df = 1 for balance).

Supplementary Table 3. Frequencies of *IFNL3* and *IFNL4* haplotypes concerning spontaneous HCV elimination (cases) and persistent HCV infection (controls)

| SNPs | Haplotype | Haplotype Frequencies | Case, Control  Frequencies | Chi-Square | P Value | P_corr_  Value^†^ | OR (95%CI)^‡^, P Value | OR (95%CI)^§^, P Value |
| --- | --- | --- | --- | --- | --- | --- | --- | --- |
| s12980275_rs4803217 | A_C | 0.582 | 0.689, 0.502 | 11.259 | 8.0E-4 | **0.0040** | **2.238 (1.406 - 3.562), 0.0006** | **Reference** |
|  | G_A | 0.326 | 0.248, 0.384 | 6.532 | 0.0106 | **0.0420** | **0.531 (0.325 - 0.865), 0.0105** | **0.469 (0.284 - 0.773), 0.0027** |
|  | G_C | 0.062 | 0.046, 0.074 | 1.052 | 0.3052 | 0.7270 | 0.560 (0.210 - 1.149), 0.2428 | 0.420 (0.155 - 1.139), 0.0808 |
|  | A_A | 0.030 | 0.016, 0.040 | 1.505 | 0.2199 | 0.6170 | 0.377 (0.077 - 1.847), 0.2119 | 0.279 (0.057 - 1.382), 0.0970 |
| rs4803217_rs12979860 | C_C | 0.621 | 0.713, 0.554 | 8.438 | 0.0037 | **0.0110** | **1.999 (1.247 - 3.205), 0.0038** | **Reference** |
|  | A_T | 0.340 | 0.250, 0.407 | 8.594 | 0.0034 | **0.0110** | **0.484 (0.298 - 0.788), 0.0033** | **0.477 (0.292 - 0.779), 0.0029** |
|  | C_T | 0.022 | 0.022, 0.023 | 0.000 | 0.9937 | 1.0000 | 1.015 (0.223 - 4.613), 0.9846 | 0.789 (0.172 -3.616), 0.7594 |
|  | A_C | 0.016 | 0.015, 0.017 | 0.023 | 0.8796 | 0.9890 | 0.901 (0.148 - 5.467), 0.9093 | 0.701 (0.115 - 4.188), 0.6992 |
| rs12979860_rs368234815 | C_TT | 0.621 | 0.713, 0.553 | 8.453 | 0.0036 | **0.0050** | **1.975 (1.231 - 3.168), 0.0045** | **Reference** |
|  | T_∆G | 0.334 | 0.235, 0.407 | 10.368 | 0.0013 | **0.0010** | **0.443 (0.270 - 0.726), 0.0011** | **0.449 (0.273 - 0.739), 0.0015** |
|  | T_TT | 0.029 | 0.037, 0.023 | 0.580 | 0.4462 | 0.8800 | 1.708 (0.450 - 6.486), 0.4265 | 1.314 (0.343 - 5.041), 0.6893 |
|  | C_∆G | 0.016 | 0.015, 0.017 | 0.024 | 0.8768 | 0.9860 | 1.351 (0.188 - 9.716), 0.7643 | 1.052 (0.145 - 7.617), 0.9603 |
| rs368234815_rs8099917 | TT_T | 0.623 | 0.732, 0.543 | 11.811 | 6.0E-4 | **0.0020** | **2.376 (3.849 - 11.47), 0.0004** | **Reference** |
|  | ∆G_G | 0.183 | 0.107, 0.239 | 9.170 | 0.0025 | **0.0060** | **0.371 (0.194 - 0.710), 0.0021** | **0.321 (0.166 - 0.624), 0.0005** |
|  | ∆G_T | 0.167 | 0.143, 0.185 | 0.970 | 0.3247 | 0.6970 | 0.729 (0.395 - 1.344), 0.3097 | 0.564 (0.302 - 1.056), 0.0715 |
|  | TT_G | 0.027 | 0.018, 0.033 | 0.626 | 0.4289 | 0.8010 | 0.450 (0.189 - 2.263), 0.3201 | 0.337 (0.066 - 1.709), 0.1697 |
| rs12980275_rs4803217_rs12979860 | A_C_C | 0.572 | 0.674, 0.497 | 10.091 | 0.0015 | **0.0080** | **2.116 (1.330 - 3.369), 0.0015** | **Reference** |
|  | G_A_T | 0.320 | 0.242, 0.379 | 6.767 | 0.0093 | **0.0490** | **0.523 (0.319 - 0.857), 0.0095** | **0.471 (0.284 - 0.782), 0.0033** |
|  | G_C_C | 0.049 | 0.039, 0.057 | 0.573 | 0.4489 | 0.9850 | 0.667 (0.222 - 1.998), 0.4663 | 0.500 (0.164 - 1.521), 0.2145 |
|  | A_A_T | 0.020 | 0.008, 0.028 | 1.618 | 0.2034 | 0.8350 | 0.266 (0.031 - 2.306), 0.1977 | 0.200 (0.023 - 1.747), 0.1080 |
|  | G_C_T | 0.012 | 0.008, 0.016 | 0.442 | 0.5064 | 0.9960 | 0.449 (0.046 - 4.363), 0.478 | 0.333 (0.034 - 3.266), 0.3225 |
|  | A_C_T | 0.010 | 0.015, 0.007 | 0.537 | 0.4636 | 0.9900 | 2.742 (0.246 - 30.580), 0.3929 | 2.000 (0.178 - 22.460), 0.5669 |
|  | A_A_C | 0.010 | 0.008, 0.011 | 0.059 | 0.8079 | 1.0000 | 0.677 (0.061 - 7.546), 0.7494 | 0.500 (0.045 - 5.615), 0.5669 |
| rs4803217_rs12979860_rs368234815 | C_C_TT | 0.612 | 0.705, 0.543 | 8.690 | 0.0032 | **0.0050** | **2.080 (0.282 - 3.376), 0.0028** | **Reference** |
|  | A_T_∆G | 0.315 | 0.220, 0.385 | 9.855 | 0.0017 | **0.0040** | **0.454 (0.274 - 0.753), 0.0020** | **0.446 (0.268 - 0.744), 0.0018** |
|  | A_T_TT | 0.025 | 0.030, 0.022 | 0.182 | 0.6699 | 1.0000 | 1.359 (0.334 - 5.540), 0.6672 | 1.042 (0.253 - 4.285), 0.9549 |
|  | C_T_∆G | 0.019 | 0.015, 0.022 | 0.213 | 0.6448 | 1.0000 | 0.669 (0.1207 - 3.711), 0.6437 | 0.521 (0.0.093 - 2.911), 0.4500 |
| rs12979860_rs368234815_rs8099917 | C_TT_T | 0.614 | 0.712, 0.541 | 9.623 | 0.0019 | **0.0020** | **2.039 (1.271 - 3.272), 0.0029** | **Reference** |
|  | T_∆G_G | 0.184 | 0.105, 0.243 | 9.848 | 0.0017 | **0.0020** | **0.349 (0.183 - 0.668), 0.0011** | **0.321 (0.166 - 0.622), 0.0005** |
|  | T_∆G_T | 0.149 | 0.130, 0.164 | 0.714 | 0.3980 | 1.0000 | 0.773 (0.411 - 1.454), 0.4234 | 0.619 (0.324 - 1.182), 0.1439 |
|  | T_TT_G | 0.018 | 0.019, 0.017 | 0.023 | 0.8783 | 1.0000 | 1.346 (0.267 - 6.776). 0.7177 | 1.031 (0.203 - 5.235), 0.9707 |
|  | C_∆G_T | 0.016 | 0.015, 0.017 | 0.025 | 0.8735 | 1.0000 | 0.891 (0.147 - 5.407), 0.8997 | 0.687 (0.112 - 4.205), 0.6832 |
|  | T_TT_T | 0.011 | 0.018, 0.006 | 1.072 | 0.3006 | 0.9970 | 2.701 (0.242 - 30.121), 0.4005 | 2.062 (0.184 - 23.124), 0.5490 |
| rs12980275_rs4803217_rs12979860_  rs368234815 | A_C_C_TT | 0.566 | 0.668, 0.491 | 9.996 | 0.0016 | **0.0230** | **2.199 (1.368 - 3.535), 0.0012** | **Reference** |
|  | G_A_T_∆G | 0.295 | 0.212, 0.357 | 7.884 | 0.0050 | **0.0300** | **0.478 (0.286 - 0.799), 0.0044** | **0.435 (0.257 - 0.735), 0.0017** |
|  | G_C_C_TT | 0.046 | 0.038, 0.052 | 0.384 | 0.5357 | 1.0000 | 0.741 (0.242 - 2.265), 0.6730 | 0.550 (0.177 - 1.704), 0.3272 |
|  | G_A_T_TT | 0.025 | 0.030, 0.022 | 0.184 | 0.6675 | 1.0000 | 1.362 (0.334 - 5.552), 0.6672 | 0.989 (0.140 - 4.078), 0.988 |
|  | A_A_T_∆G | 0.020 | 0.008, 0.028 | 1.630 | 0.2017 | 0.7160 | 0.196 (0.031 - 2.294), 0.1949 | 1.198 (0.023 - 1.728), 0.1052 |
|  | G_C_T_∆G | 0.012 | 0.007, 0.016 | 0.466 | 0.4950 | 1.0000 | 0.446 (0.046 - 4.341), 0.4742 | 0.330 (0.034 - 3.231), 0.3173 |
| rs4803217_rs12979860_rs368234815_  rs8099917 | C_C_TT_T | 0.604 | 0.704, 0.530 | 9.897 | 0.0017 | **0.0010** | **2.190 (1.338 - 3.584), 0.0017** | **Reference** |
|  | A_T_∆G_G | 0.176 | 0.102, 0.230 | 8.887 | 0.0029 | **0.0020** | **0.379 (0.197 - 0.730), 0.0029** | **0.340 (0.175 - 0.663), 0.0011** |
|  | A_T_∆G_T | 0.139 | 0.118, 0.155 | 0.874 | 0.3499 | 0.8610 | 0.702 (0.363 - 1.355), 0.2897 | 0.563 (0.188 - 1.103), 0.0916 |
|  | A_T_TT_G | 0.016 | 0.015, 0.016 | 0.014 | 0.9055 | 1.0000 | 0.891 (0.147 - 5.411), 0.8998 | 0.681 (0.111 - 4.165), 0.6753 |
|  | C_T_∆G_T | 0.012 | 0.015, 0.009 | 0.206 | 0.6498 | 0.9900 | 1.344 (0.187 - 9.672), 0.7684 | 1.021 (0.141 - 7.398), 0.9837 |

^†^ P-value calculated using the permutation test and a total of 1,000 permutations.

^‡^ All other haplotypes pooled together were used as the reference.

^§^ The most common haplotype was used as the reference

Comments to Supplementary Table 3 showing haplotypes of 2 – 4 SNPs. If all haplotypes other than the tested one were pooled together and used as the reference, spontaneous HCV clearance was positively associated with haplotypes composed of at least two major alleles but without any variant allele of tested *IFNL3*/*IFNL4* SNPs. The strongest association with spontaneous HCV resolution was revealed for the haplotype rs368234815TT_rs8099917T (OR 2.376, 95% CI 3.849 - 11.467, P = 0.0004). The most predictable haplotype, associated with lower probability of spontaneous HCV clearance, was rs12979860T_rs368234815∆G_rs8099917G composed of three variant alleles (OR 0.349, 95% CI 0.183 - 0.688, P = 0.001).

Supplementary Table 4. Frequencies of *IFNL3* and *IFNL4* haplotypes concerning spontaneous HCV elimination (cases) and persistent HCV infection (controls) in European HD patients

|  |  | **Number of haplotypes** | **Frequency of haplotypes** |  |
| --- | --- | --- | --- | --- |
| **SNPs** | **Haplotype** | **Cases, Controls** | **Cases, Controls** | **OR (95%CI), P Value** |
| **rs368234815_rs12979860** | ∆G_T | 32, 75 | 0.941, 0.962 | **Reference** |
|  | ∆G_C | 2, 3 | 0.059, 0.038 | 1.563 (0.249 - 9.808), 0.6314 |
| **rs368234815_rs8099917** | ∆G_G | 15, 44 | 0.441, 0.564 | **Reference** |
|  | ∆G_T | 19, 34 | 0.559, 0.436 | 1.639 (0.728 - 3.691), 0.2309 |
| **rs368234815_rs4803217** | ∆G_A | 31, 72 | 0.912, 0.923 | **Reference** |
|  | ∆G_C | 3, 6 | 0.088, 0.077 | 1.161 (0.273 - 4.945), 0.8395 |
| **rs368234815_rs12980275** | ∆G_G | 31, 70 | 0.912, 0.897 | **Reference** |
|  | ∆G_A | 3, 8 | 0.088, 0.103 | 0.847 (0.210 - 3.410), 0.8148 |

Supplementary Figure 1A. Chromosomal localization of tested *IFNL3* and *IFNL4* polymorphisms on chromosome 19q13.2


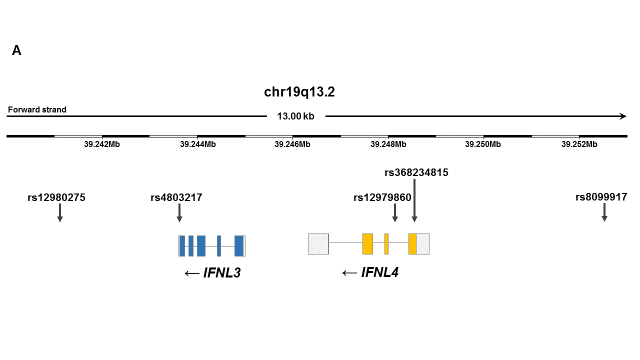


Supplementary Fig. 1B.


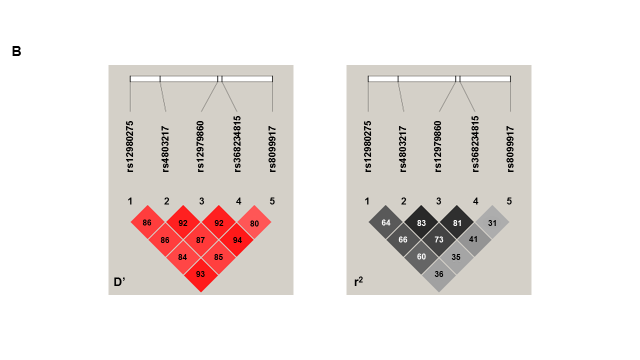


Supplementary Fig. 2. The *IFNL4* rs368234815 polymorphism and mortality due to neoplasms in anti-HCV positive HD patients

1. Stratification by *IFNL4* rs368234815 genotypes

P-value for HWE = 1.000

log-rank P = 0.010


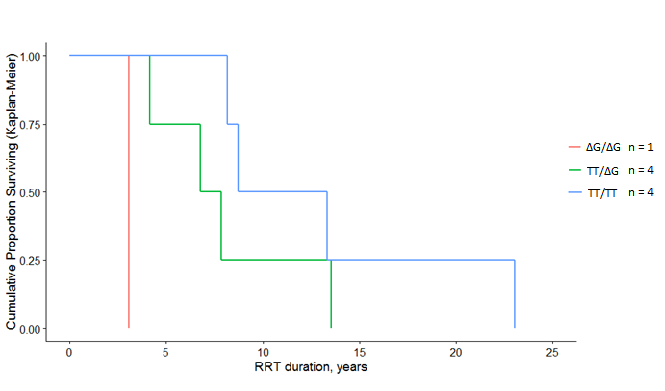


1. A recessive mode of inheritance

log-rank P = 0.005


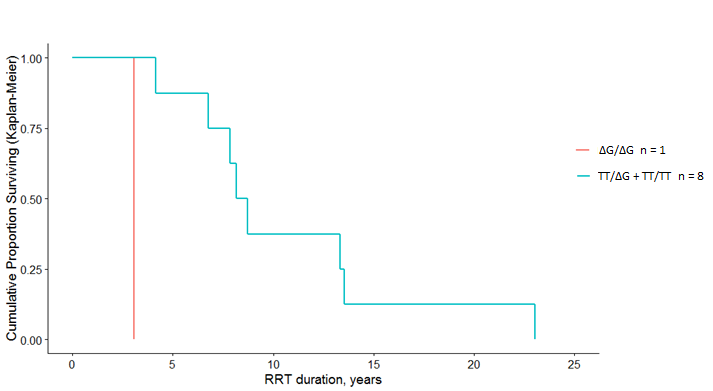

Supplement: Supplementary file 1 — Additional file 1: Supplementary data for polymorphism rs368234815 of interferon lambda 4 gene and spontaneous clearance of hepatitis C virus in haemodialysis patients. Supplementary material contains genotyping conditions for identifying tested polymorphisms, results of Hardy–Weinberg equilibrium, and haplotype analysis. Figures show chromosomal localization of tested IFNL3/IFNL4 polymorphisms and the Kaplan-Meier cumulative proportion surviving for haemodialysis patients with neoplasms. [file 12879_2021_5777_MOESM1_ESM.docx]
